# Adiponectin Modulates Oxidative Stress-Induced Autophagy in Cardiomyocytes

**DOI:** 10.1371/journal.pone.0068697

**Published:** 2013-07-19

**Authors:** Eric E. Essick, Richard M. Wilson, David R. Pimentel, Masayuki Shimano, Simoni Baid, Noriyuki Ouchi, Flora Sam

**Affiliations:** 1 Whitaker Cardiovascular Institute, Boston University School of Medicine, Boston, Massachusetts, United States of America; 2 Cardiovascular Section and Evans Department of Medicine, Boston University School of Medicine, Boston, Massachusetts, United States of America; UAE University, Faculty of Medicine & Health Sciences, United Arab Emirates

## Abstract

Diastolic heart failure (HF) i.e., “HF with preserved ejection fraction” (HF-preserved EF) accounts for up to 50% of all HF presentations; however there have been no therapeutic advances. This stems in part from an incomplete understanding about HF-preserved EF. Hypertension is the major cause of HF-preserved EF whilst HF-preserved EF is also highly associated with obesity. Similarly, excessive reactive oxygen species (ROS), i.e., oxidative stress occurs in hypertension and obesity, sensitizing the heart to the renin-angiotensin-aldosterone system, inducing autophagic type-II programmed cell death and accelerating the propensity to adverse cardiac remodeling, diastolic dysfunction and HF. Adiponectin (APN), an adipokine, mediates cardioprotective actions but it is unknown if APN modulates cardiomyocyte autophagy. We tested the hypothesis that APN ameliorates oxidative stress-induced autophagy in cardiomyocytes. Isolated adult rat ventricular myocytes were pretreated with recombinant APN (30µg/mL) followed by 1mM hydrogen peroxide (H_2_O_2_) exposure. Wild type (WT) and APN-deficient (APN-KO) mice were infused with angiotensin (Ang)-II (3.2mg/kg/d) for 14 days to induced oxidative stress. Autophagy-related proteins, mTOR, AMPK and ERK expression were measured. H_2_O_2_ induced LC3I to LC3II conversion by a factor of 3.4±1.0 which was abrogated by pre-treatment with APN by 44.5±10%. However, neither H_2_O_2_ nor APN affected ATG5, ATG7, or Beclin-1 expression. H_2_O_2_ increased phospho-AMPK by 49±6.0%, whilst pretreatment with APN decreased phospho-AMPK by 26±4%. H_2_O_2_ decreased phospho-mTOR by 36±13%, which was restored by APN. ERK inhibition demonstrated that the ERK-mTOR pathway is involved in H_2_O_2_-induced autophagy. Chronic Ang-II infusion significantly increased myocardial LC3II/I protein expression ratio in APN-KO vs. WT mice. These data suggest that excessive ROS caused cardiomyocyte autophagy which was ameliorated by APN by inhibiting an H_2_O_2_-induced AMPK/mTOR/ERK-dependent mechanism. These findings demonstrate the anti-oxidant potential of APN in oxidative stress-associated cardiovascular diseases, such as hypertension-induced HF-preserved EF.

## Introduction

Diastolic heart failure (HF) i.e., HF with *preserved* ejection fraction (HF-preserved EF) accounts for ~50% of all clinical HF presentations [1]; but unlike systolic HF i.e., HF with *reduced* ejection fraction (HF-reduced EF), there are no evidenced-based therapies [2]. Although hypertension [3–5] and obesity are both commonly associated with HF-preserved EF [6,7], there remains an incomplete mechanistic understanding about HF-preserved EF. Recently we showed that low adiponectin levels increased the propensity to diastolic HF and diastolic dysfunction in an experimental murine model [8]. Adiponectin (APN), an adipocyte-derived cytokine (adipokine), modulates cardiac dysfunction by its interaction with several intracellular signaling pathways [9]. Hypoadiponectinemia reflects increased cardiovascular risk and inflammation, in conditions such as hypertension, coronary artery disease, obesity and insulin resistance [10–12]. However, in humans with systolic HF (or HF-reduced EF) [13–15], APN levels are elevated and correlate with HF symptoms [16], disease severity and mortality [13,14]. Despite these conflicting data, APN levels are also elevated in an *in vivo* model of premature aging and oxidative stress [17,18], suggesting that APN levels are increased in an attempt to mitigate the deleterious effects of accelerated aging [17]. Thus in HF-reduced EF, hyperadiponectinemia may reflect an attempt to mitigate pro-inflammatory or impaired metabolic states, demonstrating a balance between protective and harmful pathways. Thus the interaction between factors secreted by adipocytes and cardiomyocytes, in cardiac diseases such as HF-preserved EF, requires further investigation.

Excessive reactive oxygen species (ROS) is seen in conditions like hypertension, obesity and HF-preserved EF and overwhelms antioxidant defenses leading to a state of oxidative stress [19,20]. Although ROS are generated in a highly regulated manner in cardiomyocytes [21]; excessive ROS causes adverse left ventricular (LV) remodeling resulting in cell death [22], contractile dysfunction and ultimately clinical HF [23]. NADPH oxidase is the major source of ROS in the heart and although superoxide is the first moiety generated by NADPH oxidase, the signaling effects of ROS appear to be mediated by the more stable and diffusible hydrogen peroxide (H_2_O_2_). As we and others have shown, physiologically increased levels of H_2_O_2_ (1-100µM) activates ROS-mediated signaling pathways including MAPK members and NF-κB [24,25] and induces compensated left ventricular hypertrophy (LVH) [26]. We recently showed that APN mediates cardioprotective effects against physiologically increased ROS (1µM H_2_O_2_) in cardiomyocytes by regulating an AMPK/ERK/NF-κB signaling axis [25]. However, pathophysiological levels of ROS causes both apoptosis [22,27] and autophagy [28,29] in the cardiovascular system, eventually impairing myocardial function [30].

Although, autophagy is upregulated during periods of stress, such as cell starvation it has a “Janus-like” role in degenerative diseases such as Alzheimer’s and cancer, where it might be both deleterious or protective [17,31,32]. In chronic cardiac stress, such as HF and hypertension, oxidative stress induces maladaptive autophagy [33], possibly resulting in compensated LVH transitioning to decompensated HF [34,35]. Despite pathophysiological levels of H_2_O_2_ increasing oxidative stress and cardiomyocyte apoptosis [22], little is known about the effects of APN on pathophysiological levels of ROS-induced autophagy. We thus sought to test the hypothesis that the cardioprotective actions of APN extends to modulating pathophysiological levels of ROS-induced autophagy in cardiomyocytes and to define the intracellular signaling pathways involved.

## Materials and Methods

All of the animals were treated according to the guidelines of the Guide for the Care and Use of Laboratory Animals (United States National Institutes of Health). The Institutional Animal Care and Use Committee at Boston University School of Medicine approved all study procedures related to handling and surgery of mice and rats. All efforts were made to minimize the number of animals used and their suffering***.***

### Chemicals and reagents

H_2_O_2_ (30% w/w), trichloroacetic acid, thiazolyl blue tetrazolium bromide, Bafilomycin A1, and Compound C (AMPK inhibitor) were purchased from Sigma-Aldrich (St. Louis, MO). The MEK1/2 inhibitor U0126 was purchased from Cell Signaling Technology (Danvers, MA). Recombinant APN was prepared as previously described [25]. Mouse APN (amino acids 15-247) was cloned into the bacterial expression vector pTrcHisB (Amersham). The histidine-tagged proteins were purified using nickel ion-agarose column, mono Q column, and for removal of lipopolysaccharide, Detoxi-Gel Affinity Pak column (Pierce Scientific, Rockford, IL) [25].

### Isolation and treatment of adult rat cardiac myocytes

Adult rat ventricular myocytes (ARVM) were isolated as follows. Briefly, ARVM (90-95% purity) were harvested from adult male Sprague-Dawley rats (approximately 200-220g) and plated non-confluently on laminin-coated (1µg/cm^2^; Invitrogen, Carlsbad, CA) plastic culture dishes (Fisher Scientific, Pittsburgh, PA) at a density of 30-50 cells/mm^2^. Cells were maintained at 37^°^ C prior to treatment, in Dulbecco’s Modified Eagle Medium (DMEM, Invitrogen) containing, 2mg/mL BSA, 2mmol/L L-carnitine, 5mmol/L creatinine, 5mmol/L taurine (Sigma-Aldrich), 100IU/mL penicillin, and 10g/mL streptomycin (Invitrogen). ARVM were subjected to various concentrations of H_2_O_2_ (Sigma) and the final concentration of 1mM was used, which has been described by others to induce cell death [22]. In some experiments ARVM were pre-treated with recombinant APN (30µg/mL) for 18hrs prior to H_2_O_2_ treatment.

### *In vivo* murine model

Male WT and APN-KO mice in a C57/BL6 background were used as previously described [25]. The Institutional Animal Care and Use Committee at Boston University School of Medicine approved all study procedures related to the handling and surgery of the mice. WT and APN-KO were subjected to subcutaneous Ang-II (3.2mg/kg/d) or saline infusion using an implanted osmotic minipump (Durect Corporation, Cupertino, CA). Tail cuff blood pressure (SBP), non-invasive heart rate (HR), morphology and echocardiography measurements were performed as previously described [25]. Fourteen days after Ang-II infusion, mice were sacrificed, hearts were dissected and the LV was snap-frozen in liquid nitrogen.

### Echocardiography determinants for LV dimensions

Transthoracic echocardiography was performed in conscious mice using an Acuson Sequoia C-256 echocardiography machine and a 15-MHz probe as previously described [25,60].

### MTT Assay

The MTT (3-[4,5-dimethylthiazol-2-yl]-2,5-diphenyl tetrazolium bromide) assay is an index of cell viability, and used to determine the effects of H_2_O_2_ on cell viability. It measures the enzymatic activity of mitochondrial reductase enzymes, which reduce MTT (from a yellow water soluble dye) to insoluble formazan (dark blue). These enzymes are active during times of cell stress and altered states of cellular metabolism and thus serve as an appropriate method for the assessment of viability. ARVM were pretreated with or without 30µg/mL APN (18hrs) and then treated with 1mM H_2_O_2_ for 90min in phenol-free DMEM. 50µL MMT/mL was added for the last 1hr and protected from light. Following the removal of media, DMSO was added to solubilize the cells. Sorensen’s buffer was added to DMSO, and 100µL was transferred to a 96-well plate. Absorbance was measured at a wavelength of 570nm (A_570_) using a plate reader, and data is presented as a ratio to control group, measured in arbitrary units. Cell viability of the experimental group was determined as a percentage of the reading of the control group.

### Adenovirus infection

In some experiments, ARVM were infected with adenoviral constructs encoding dominant-negative Akt1 (Ad-dnAkt) with a hemagglutinin (HA) tag or Ad-β-gal at 10 moi with H_2_O_2_. In addition, viral transfection with green fluorescent protein (GFP)-labeled LC was performed as follows. GFP-LC3 adenoviral infection was used to quantify autophagosome formation. In order to visually assess the presence or absence of autophagosomes, cells were transfected with a GFP-microtubule-associated protein light chain 3 GFP-LC3 virus, achieving at least 40% transfection efficiency. ARVMs were transfected at a multiplicity of infection (moi) of 10 for 4hrs. Cell media was changed and cells were then allowed to incubate overnight for maximal GFP-LC3 expression. At the time of experiment, cells were treated with the vacuolar H^+^-ATPase inhibitor Bafilomycin-A1 (50nM) in addition to their respective treatments in order to inhibit autophagolysosome degradation. Following treatment exposure, media was removed and cells were fixed in 4% paraformaldehyde in PBS (pH 7.4) at room temperature for 15min. Cells were stored at 4^°^ C in PBS and observed under oil at 60x magnification (Nikon Diaphot 300). To quantify autophagosome formation, the number of GFP-LC3 puncta per cell was counted in multiple fields and averaged. Experiments were replicated 4 times.

### Western Blot Analysis

Isolated ARVMs were subjected to 12% SDS-PAGE and Western blotting for protein expression. Following H_2_O_2_ treatment, ARVM were lysed, scraped, and collected in cold lysis buffer, and total protein concentration was measured by Bradford assay (Bio-Rad Laboratories, Hercules, CA). In the AMPK experiments Compound C was purchased from Sigma Aldrich, St. Louis, MO. Samples were prepared and subjected to SDS-PAGE (12% tris-glycine gels, Lonza, Rockland, ME) and Western blotting. Membranes were probed for autophagic proteins with the following antibodies: polyclonal anti-LC3 (1:2500); anti-ATG5 (1:2500); anti-ATG7 (1:2500) (Novus Biologicals, Littleton CO), polyclonal anti-beclin-1 (1:1000) (Cell Signaling Technology, Danvers MA), and polyclonal phospho-mTOR (Ser2448) antibody (1:1000) and phospho-Akt (Ser-473) (1:1000) (Cell Signaling Technology). In addition, membranes were probed for phosphorylated and total ERK and phosphorylated and total AMPK using the following antibodies: monoclonal against p-p44/42 (ERK) (1:1000), polyclonal total p44/42 ERK (1:1000), polyclonal p-AMPKα (Thr172) (1:1000), and polyclonal AMPKα (Cell Signaling Technology). Anti-GAPDH monoclonal antibody was used as a loading control house keeping protein (Thermo Scientific. Rockford, IL). Membranes were then probed with either goat anti-rabbit or goat anti-mouse horseradish peroxidase conjugated secondary antibodies (1:5000) (Santa Cruz Biotechnology, Santa Cruz, CA). Blots were detected with ECL^TM^ Western Blotting Detection Reagent (Amersham), and chemiluminescence was quantified by densitometry using ImageJ measuring software (National Institutes of Health). Protein expression was normalized for equal protein loading, and data is expressed in arbitrary units relative to control.

### RNA isolation and RT-PCR to assess gene expression

Akt, mTOR and GAPDH mRNA levels from isolated ARVMs were quantified by RT-PCR. Following experimental procedures, total RNA was extracted from ARVM with Qiagen RNeasy Micro kit (Valencia, CA) according to the manufacturer’s instructions. cDNA was generated from total RNA using SuperScript® III First-Strand synthesis kit purchased from Invitrogen (Carlsbad, CA). Transcript expression levels were quantified by StepOne Plus Real-Time PCR Detection Systems (Applied Biosystems, Warrington, UK) using SYBR® Green Master Mix (Applied Biosystems, Warrington, UK). Akt and mTOR transcript levels were then adjusted relative to GAPDH expression. The PCR primers were manufactured by Integrated DNA Technologies (Coralville, IA) and the rat sequences are as follows:

Akt1 forward: 5′-ACCTCTGAGACCGACACCAG-3′,
Akt1 reverse: 5′-AGGAGAACTGGGGAAAGTGC-3′;
mTOR forward: 5′- GCTTATCAAGCAAGCGACATCTCA-3′,
mTOR reverse: 5′- TCCACTGGAAGCACAGACCAAG-3′,
GAPDH forward: 5’-CTGCACCACCAACTGCTTAG-3’,
GAPDH reverse: 5’-CTTCTGAGTGGCAGTGATGG-3’.

### Statistical analysis

All data is expressed as means ± SEM; differences among multiple conditions were determined by ANOVA followed by a paired *t*-test with the Bonferroni correction for multiple comparisons. *p* values <0.05 were considered significant.

## Results

### APN attenuated H_2_O_2_-mediated loss of cell viability

**Figure 1:**
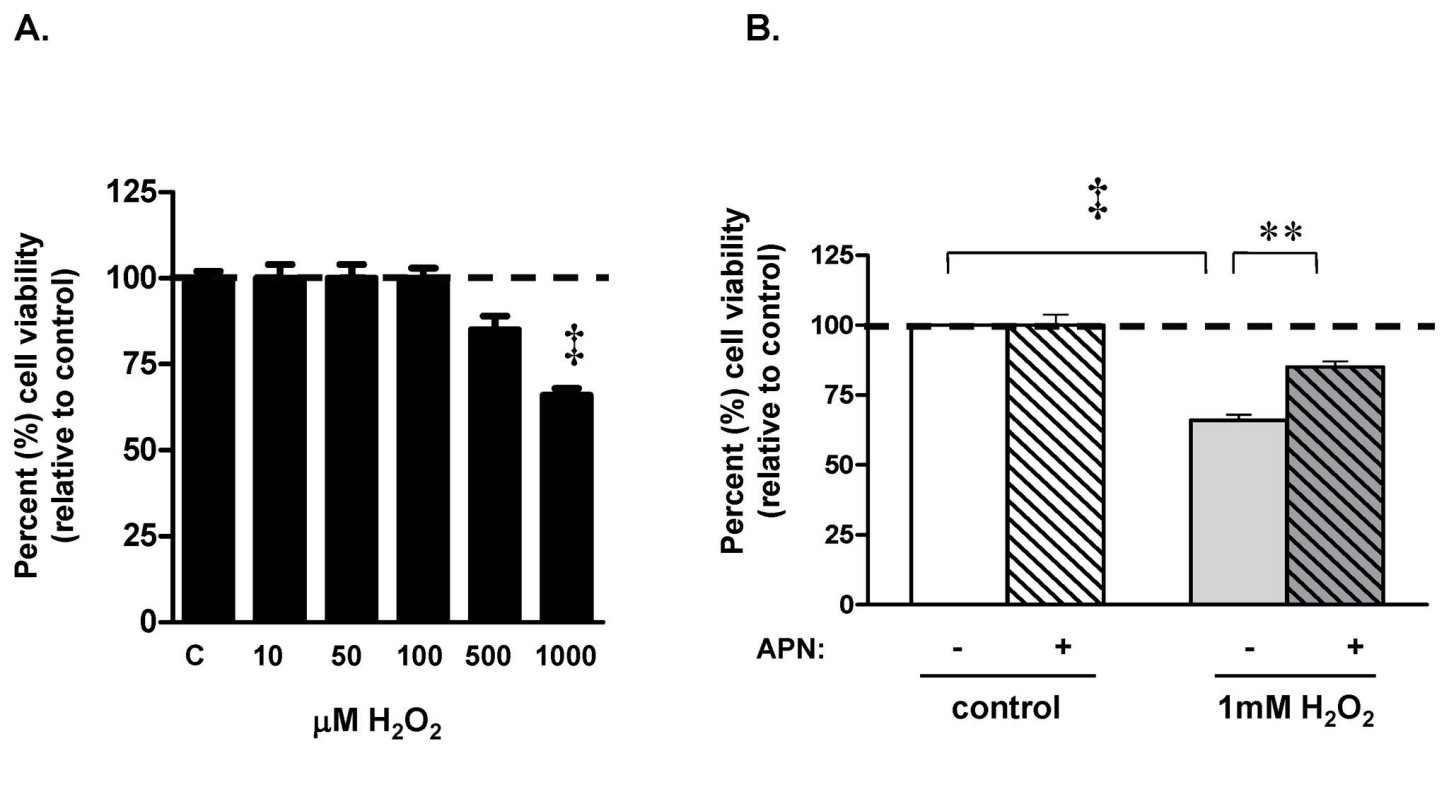
H_2_O_2_ decreased cell viability. (**A**) Myocytes were exposed to various concentrations of H_2_O_2_ for 90 min and viability was assessed by MTT uptake. Data are the mean from three experiments. ARVM were exposed to H_2_O_2_ in concentrations ranging from 10 to 1000 µM H_2_O_2_. (**B**) Adult cardiomyocytes treated with 1mM H_2_O_2_ resulted in a 34±2% decrease in viability (‡p<0.001 vs. control) as measured by MTT assay. APN pretreatment (30µg/mL), before H_2_O_2_ exposure, increased cell viability by 19±4% (**p<0.01 vs. H_2_O_2_-treated).

The MTT assay was used to determine cardiomyocyte survival in response to pathophysiological oxidative stress [36]. Isolated adult rat ventricular myocytes (ARVMs) were exposed to increasing concentrations H_2_O_2_ (10-1000µM Sigma) for 30, 60 and 90 min and viability were assessed by the MTT assay. At the 90 min time point, there was no decrease in cell viability at H_2_O_2_ concentrations of 10, 50, and 100µM. Cell viability decreased slightly at H_2_O_2_ concentrations of 500µM, and was maximally at 1000µM (p<0.001 vs. control; Figure 1A). Similar to other studies [22,37], the latter concentration was shown to induce cell death. Thus H_2_O_2_ (1mM) decreased viability by 34±2% (Figure 1B). Pretreatment with APN (30µg/mL) prior to H_2_O_2_ exposure partially improved cell viability (85±2% vs. 66±2% viable) (p<0.01 vs. H_2_O_2_-treated ARVM; Figure 1), but did not completely rescue the 34% reduction in cell viability caused by H_2_O_2_ treatment.

### APN inhibited H_2_O_2_-induced LC3-II and p62 expression and autophagosome formation

**Figure 2:**
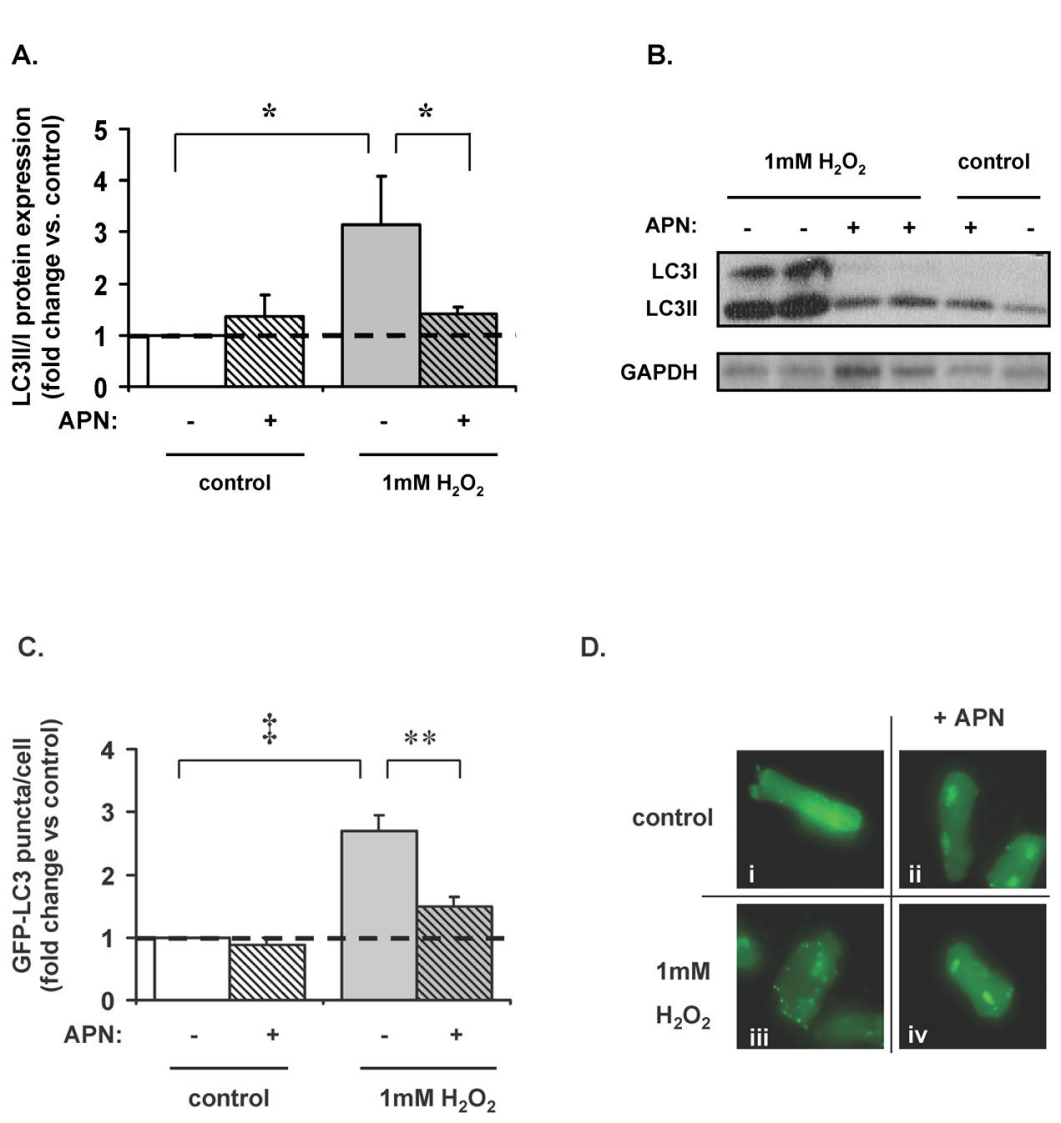
APN-attenuates H_2_O_2_-mediated autophagy in ARVM. (**A**) 1mM H_2_O_2_ increased LC3II/I protein expression ratio in ARVM by a factor of 3.4±1.0 (*p<0.05 vs. control). This was abrogated by pretreatment with APN (58±10% reduction; *p<0.05 vs. H_2_O_2_-treated cells). (**B**) Representative Western blot. (**C**) ARVMs were transfected with GFP-labeled LC3 virus (10moi) to visualize the presence of autophagosomes. 1mM H_2_O_2_ increased the number of GFP-LC3 puncta per cell by a factor of 2.7±0.2 vs. control; (‡p<0.001). Pretreatment with APN decreased this by 45±3% vs. H_2_O_2_-treated cells (**p<0.01). (**D**) Treatment with H_2_O_2_ (iii) induced the formation of the autophagosome as indicated by green puncta marking the cell perimeter vs. control (**i**). Pretreatment with APN (ii) led to a reduced number of H_2_O_2_-induced punctate autophagosomes (**iv**).

Autophagosome formation is indicative of autophagic activity. Microtubule-associated protein light chain 3 (LC3) is involved in autophagy and exists in two forms: LC3-I is the free cytosolic form, while LC3-II is conjugated to phosphotidylethanolamine (PE) and is incorporated in the autophagosome membrane [38]. LC3-II and LC3-I protein expression were measured by Western blot and the ratio of LC3-II to LC3-I protein expression was used as a measurement of autophagosome formation [39] and as an indirect indication of autophagy. In ARVM, 1mM H_2_O_2_ increased the LC3-II/LC3-I ratio by a factor of 3.4±1.0 (p<0.05 vs. control; Figure 2A). Pretreatment with APN abrogated this increased LC3-II/LC3-I ratio (p<0.05 vs. H_2_O_2_-treated cells; Figure 2A-B). To corroborate these findings, green fluorescent protein (GFP)-labeled LC3 (GFP-LC3) (10moi) expressing ARVMs were treated with 1mM H_2_O_2_ in the presence or absence of APN and analyzed under 60x oil magnification (Figure 2C-D). H_2_O_2_ increased the number of GFP-LC3 puncta per cell by a factor of 2.7±0.2 (p<0.001 vs. control), and pretreatment with APN attenuated this increase (p<0.01 vs. H_2_O_2_-treated cells). Although increased LC3-II/LC3-I ratio suggests autophagosome accumulation, increased p62 expression suggests defects in the lysosomal end of the pathway, we thus measured p62 expression in H_2_O_2_ stimulated ARVM. H_2_O_2_ (1mM) increased p62 expression (p<0.01 vs. control) and pretreatment with APN attenuated this increase (p<0.01 vs. H_2_O_2_-treated cells; Figure **S1 A-B**).

**Figure 3:**
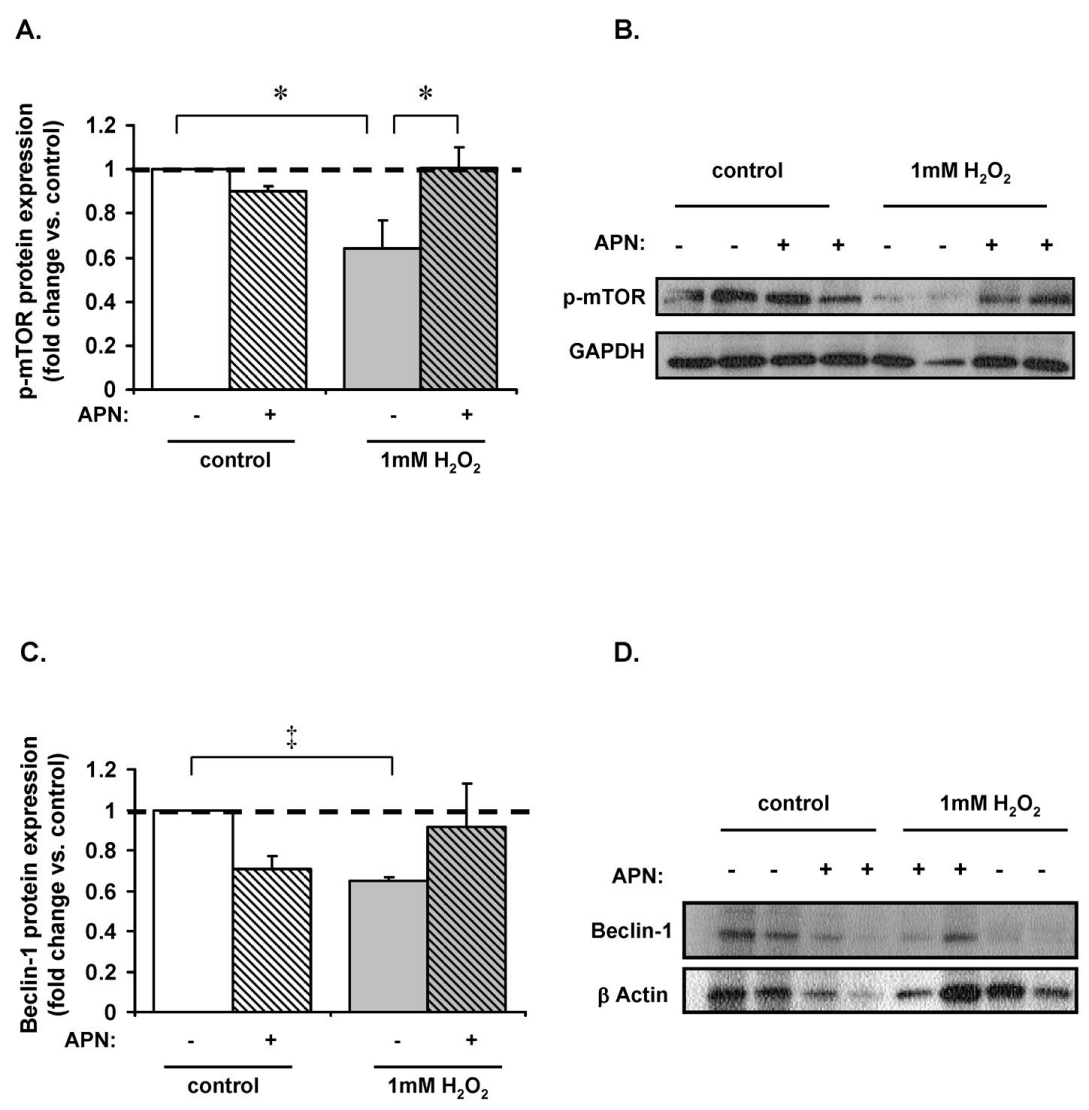
Effect on upstream regulators of autophagy. (**A**) 1mM H_2_O_2_ (15min) diminished phospho-mTOR expression by 36±13% (*p<0.05 vs. control). Pretreatment with APN restored phospho-mTOR protein expression to baseline levels (*p<0.05 vs. H_2_O_2_ treated cells). (**B**) Representative Western blot of mTOR expression. (**C**) 1mM H_2_O_2_ (15min) decreased beclin-1 protein expression by 35±2% (‡p<0.001 vs. control). Pretreatment with APN had no significant effect on beclin-1 protein expression. (**D**) Representative Western blot of beclin-1 protein expression.

APN restored loss of mTOR phosphorylation induced by H_2_O_2_: Numerous autophagic pathways converge at the mammalian target of rapamycin (mTOR), which when phosphorylated becomes a potent inhibitor of autophagy. Thus loss of mTOR phosphorylation activates autophagy [40]. In order to determine whether H_2_O_2_-mediated autophagy is occurring via an mTOR mediated pathway in our system, phospho-mTOR protein expression was measured. H_2_O_2_ (1mM) decreased phospho-mTOR expression in ARVMs by 36±13% (p<0.05 vs. control; Figure 3A-B). Conversely, APN pretreatment restored phospho-mTOR protein expression back to baseline levels (p<0.05 vs. H_2_O_2_-treated cells). Beclin-1 protein expression, an upstream promoter of autophagic induced cell death pathways [41,42], was also measured. H_2_O_2_ decreased beclin-1 protein expression by 35±2% (p<0.001 vs. control; Figure 3C-D). However, pretreatment with APN had no significant effect on beclin-1 expression. Finally, neither H_2_O_2_ nor APN significantly affected ATG5 or ATG7 expression (data not shown).

### H_2_O_2_-induced autophagy involves AMPK-mTOR phosphorylation

**Figure 4:**
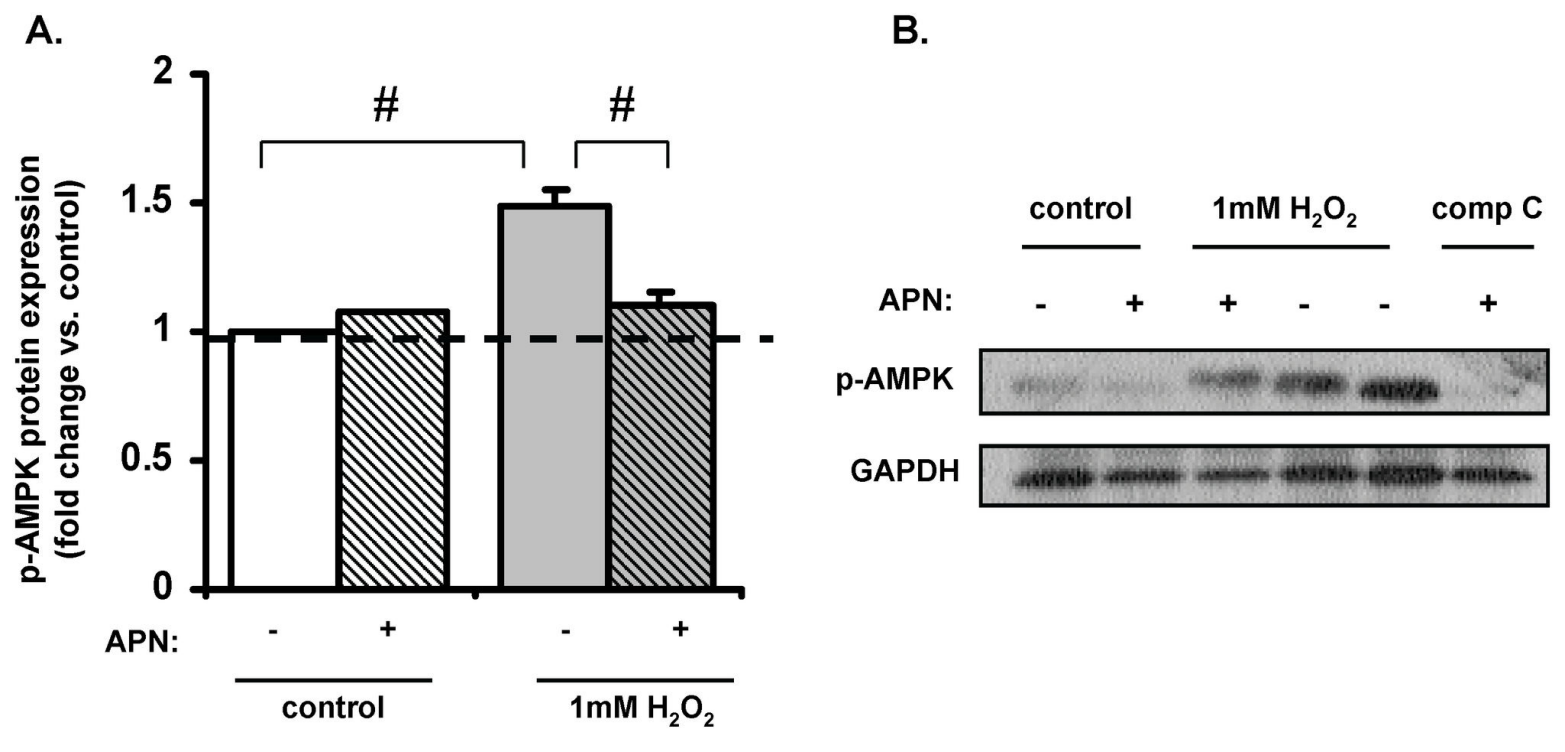
H_2_O_2_ stimulation involves AMPK/mTOR pathway. (**A**) 1mM H_2_O_2_ (15min) increased phospho-AMPK protein expression in ARVMs by 56±4% (#p<0.001 vs. control) and APN pretreatment decreased H_2_O_2_-induced phospho-mTOR expression by 28±3% (#p<0.001 vs. H_2_O_2_). (**B**) Representative Western blot of p-AMPK expression.

AMPK activation phosphorylates tuberous sclerosis complex 2 (TSC2), which inhibits mTOR and induces autophagy [43]. The role of the AMPK-mTOR dependent pathway in H_2_O_2_-induced autophagy was therefore investigated. In ARVMs treated with H_2_O_2_ (1mM), p-AMPK protein expression was increased 56±4% (p<0.001 vs. control; Figure 4A-B). In some experiments, ARVM were also treated with both compound C (50µM) and H_2_O_2_ and caused complete inhibition of p-AMPK protein expression. APN pretreatment significantly decreased H_2_O_2_-induced phospho-mTOR expression almost back to baseline (-28±3% decrease vs. H_2_O_2_). As shown in Figure 3**A-B**, H_2_O_2_ treatment also inactivated mTOR by decreasing its phosphorylation (p<0.001 vs. control), thus providing evidence for the involvement of an AMPK-mTOR dependent pathway. Although ROS (H_2_O_2_) induces autophagy in cardiomyocytes either via AMPK activation or inhibiting mTOR; APN modulates H_2_O_2_- induced autophagy only by restoring mTOR activation.

### H_2_O_2_ decreased phospho-Akt gene expression which does not involve mTOR

Since Akt mediates several processes important to cardiac adaptation including cell death inhibition and metabolism, we sought to determine if Akt was also involved in H_2_O_2_-induced autophagy. There was a 21±2% decrease in phospho-Akt gene expression in ARVMs stimulated with (1mM) H_2_O_2_ (p<0.05 vs. control). Interestingly in ARVM treated with APN alone or H_2_O_2_-simulated ARVM pretreated with APN, phospho-Akt expression was increased by comparable amounts (136±4% and 139±2%, respectively; p<0.001 vs. respective controls, Figure **S2A**). In order to examine the role of Akt in the regulation of mTOR by H_2_O_2_, ARVM were infected with a HA-tagged dominant-negative Akt (dn-Akt) or β-gal. Transduction with dn-Akt had no effect on the decreased H_2_O_2_-induced mTOR gene expression (1.0±0.01 in β-gal, 0.64±0.10 in β-gal+H _2_O_2_, 0.99±0.04 in dn-Akt, 0.65±0.06 in dn-Akt+H_2_O_2_). These data indicate that H_2_O_2_ decreased phospho-Akt expression but is not involved in H_2_O_2_-induced mTOR phosphorylation and signaling (Figure **S2 A-B**).

### ERK is required for mTOR phosphorylation in H_2_O_2_-induced autophagy

**Figure 5:**
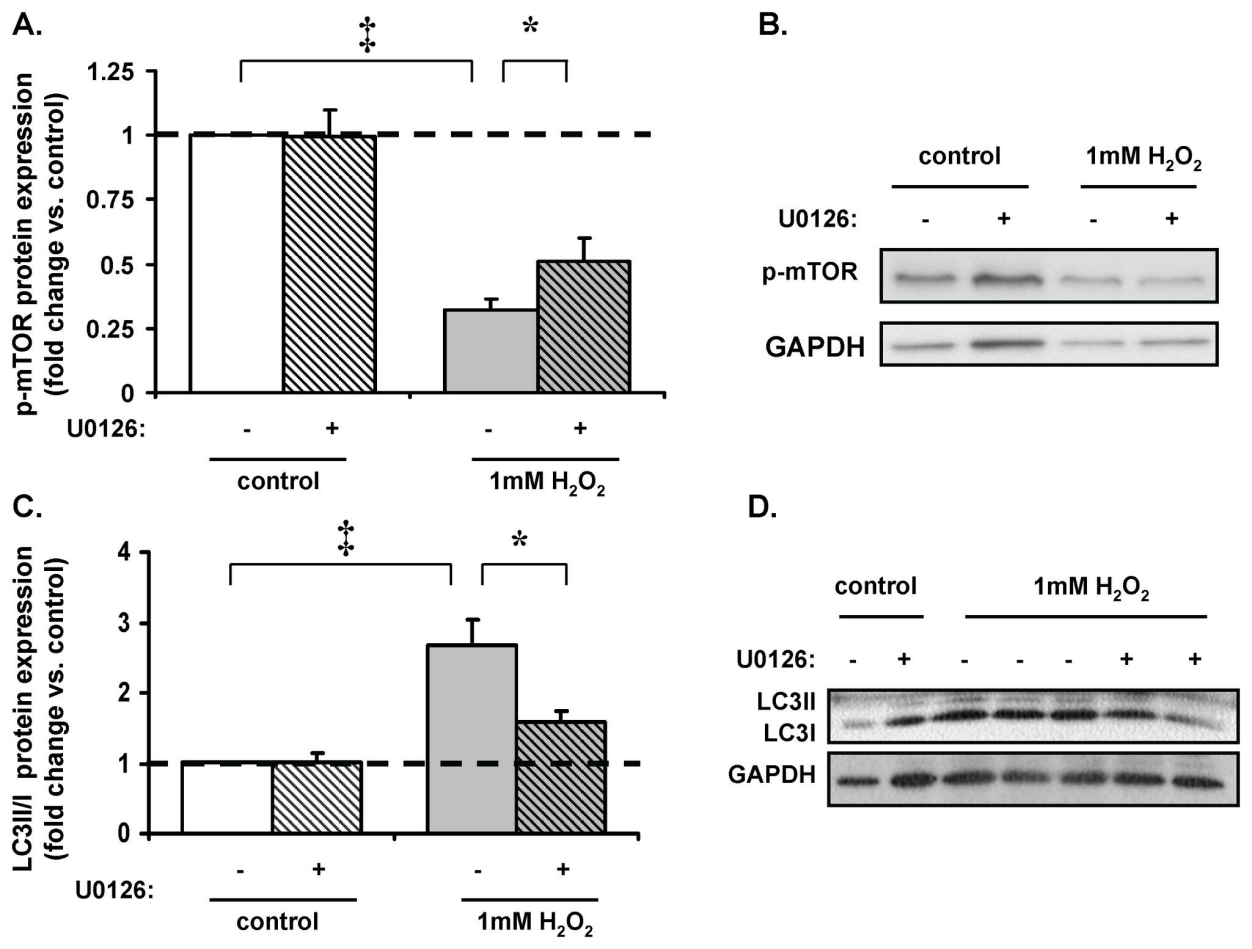
Role of ERK in H_2_O_2_ mediated autophagy. (**A**) 1mM H_2_O_2_ (15min) reduced phospho-mTOR protein expression by 32±4% (‡p<0.001 vs. control), while pretreatment with ERK inhibitor (U0126) restored phospho-mTOR protein expression by 57±22% of baseline levels (*p<0.05 vs. H_2_O_2_-treated cells). (**B**) Representative Western blot. (**C**) 1mM H_2_O_2_ increased LC3-II/LC3-I protein expression ratio by a factor of 1.7±0.4 (‡p<0.001 vs. control). ERK inhibition attenuated the H_2_O_2_-mediated increase in LC3II/I protein expression ratio by 34±8% (*p<0.05 vs. H_2_O_2_-treated cells). (**D**) Representative Western blot.

In general ERK is believed to activate mTOR [44,45]; however mTOR inhibition has been associated with increased ERK activity in response to non-starvation stress [46]. Since H_2_O_2_ increases ERK expression in cardiomyocytes [22,25], we sought to investigated the role of the ERK pathway in H_2_O_2_-induced autophagy using the MEK1/2 inhibitor U0126. H_2_O_2_ (1mM) reduced phospho-mTOR protein expression by 68±4% (p<0.001 vs. control; Figure 5A-B). However pretreatment with U0126 only partially restored phospho-mTOR protein expression by 57±22% (p<0.05 vs. H_2_O_2_-treated cells), suggesting incomplete ERK involvement. Additionally, H_2_O_2_ (1mM) increased LC3-II/LC3-I protein expression ratio by a factor of 1.68±0.36 (p<0.001 vs. control; Figure 5C-D), while ERK inhibition significantly attenuated this increase by 34±8% (p<0.05 vs. H_2_O_2_-treated cells). These results indicate that an ERK-mTOR pathway is also at least partially involved in H_2_O_2_-mediated autophagy.

### APN attenuates H_2_O_2_-induced phosphorylation of AMPK and ERK

**Figure 6:**
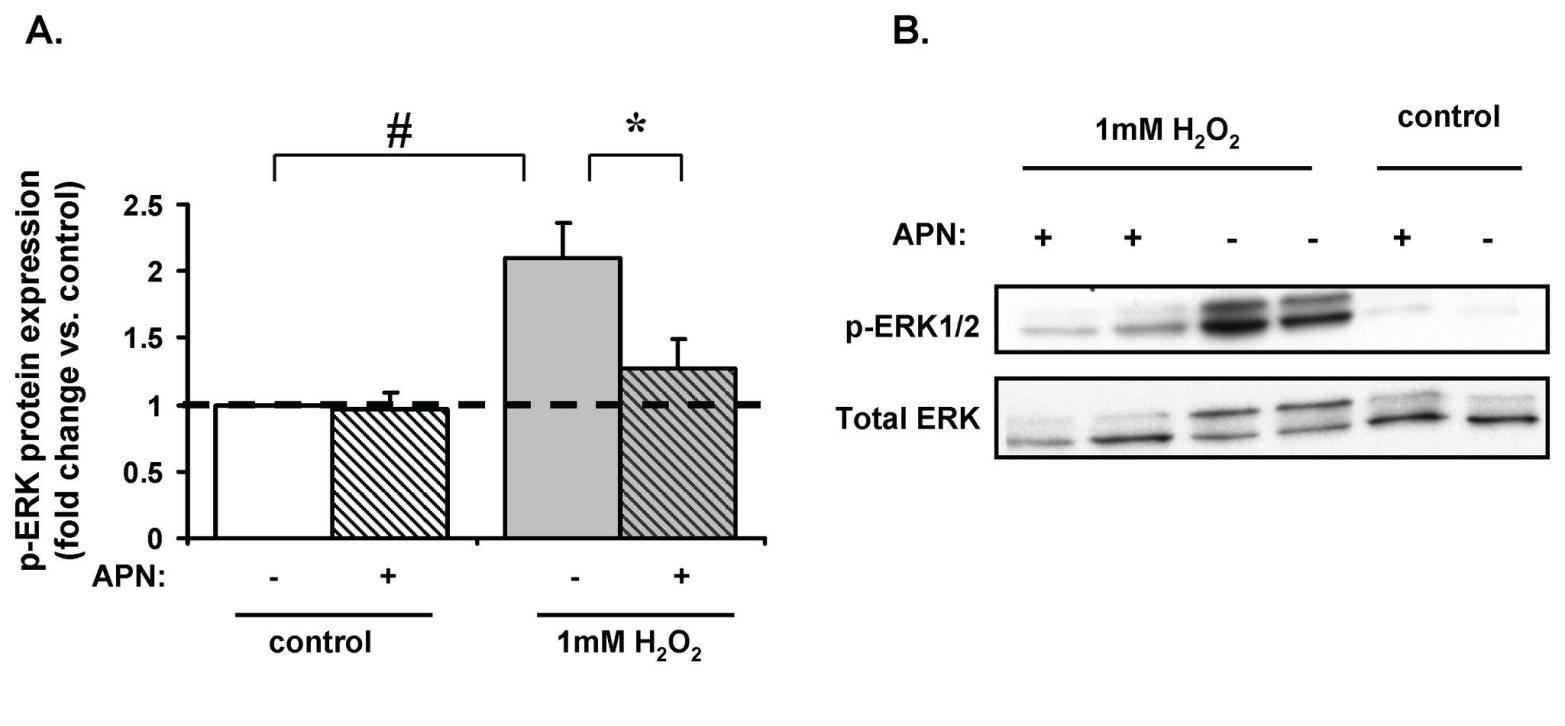
Effect of APN on H_2_O_2_ mediated increase on phospho-ERK and phospho-AMPK protein expression. (**A**) 1mM H_2_O_2_ (15min) increased phospho-ERK protein expression by a factor of 2.1±0.3 (#p<0.01 vs. control) and was attenuated by pretreatment with APN by 40±7% (*p<0.05 vs. H_2_O_2_ treated cells). (**B**) Representative Western blot.

In neonatal rat ventricular myocytes (NRVM) stimulated with norepinephrine, APN increased phospho-AMPK and inhibited phospho-ERK protein expression [47]. We thus sought to determine whether APN had direct effects on AMPK and ERK expression in response to H_2_O_2_. In ARVM APN treatment attenuated H_2_O_2_-induced phospho-ERK expression by 40±7% (p<0.05 vs. H_2_O_2_-treated cells; Figure 6A-B). Similar to our earlier finding in Figure 4**A-B**, H_2_O_2_ (1mM) increased phospho-AMPK protein expression (49±6% vs. control; p<0.01) and APN pretreatment attenuated this phospho-AMPK expression by 26±4% (p<0.01 vs. H_2_O_2_-treated cells).

We sought to determine the significance of these *in vitro* findings in an animal model of increased oxidative stress using chronic angiotensin II (Ang-II) stimulation in APN-knockout (KO) and wild-type (WT) mice [48]. As we and others showed previously, chronic Ang-II stimulation (3.2mg/kg/d for 14 days) induced hypertension and LVH [25,48] and was associated with myocardial ROS and increased expression of NADPH oxidase subunits [25,49]. Additionally, APN positively regulated AMPK expression and negatively regulated ERK expression in Ang-II infused WT hearts [25]. In this present study, we sought to determine the expression of autophagic proteins in this *in vivo* experimental model of myocardial oxidative stress from the hearts of Ang-II simulated APN-KO and WT mice. No mice died during the 14 days. At the end of 14 days blood pressure and echocardiography were obtained prior to sacrifice (Table 1).

**Table 1:** Characteristics of WT and APN-KO mice after 14 days of Ang-II infusion.

	**WT**		**APN-KO**	
	**saline**	**Ang-II**	**saline**	**Ang-II**
	*(n=3)*	(*n=6*)	*(n=3)*	*(n=6)*
HR (bpm)	561 ± 22	588 ± 15	577 ± 12	570 ± 21
SBP (mmHg)	99 ± 2	136 ± 2*	100 ± 3	140 ± 2‡
HW/BW (mg/g)	4.4 ± 0.1	6.3 ± 0.1*	4.5 ± 0.1	7.5 ± 0.1‡**
***Echoparameters***				
LVESD (mm)	1.30 ± 0.1	1.27 ± 0.1	1.33 ± 0.1	1.25 ± 0.1
LVEDD (mm)	3.0 ± 0.1	2.8 ± 0.1	3.1 ± 0.1	2.7 ± 0.1
FS (%)	55 ± 2	56 ± 3	55 ± 1	57 ± 3
IVS (mm)	0.72 ± 0.02	1.23 ± 0.04*	0.73 ± 0.02	1.45 ± 0.03‡**

Ang-II, angiotensin-II; WT, wild type; APN-KO, adiponectin knockout mice; BW, body weight; HR, heart rate; SBP, systolic blood pressure; HW, heart weight

LVEDD, left ventricular end-diastolic diameter; LVESD, left ventricular end-systolic diameter; FS, fractional shortening; IVS, interventricular septum

Results are presented as mean ± SEM.

* p<0.001 vs. WT-saline; ** p<0.01 vs. WT-Ang-II ; ‡ p<0.001 vs. APN-KO-saline

**Figure 7:**
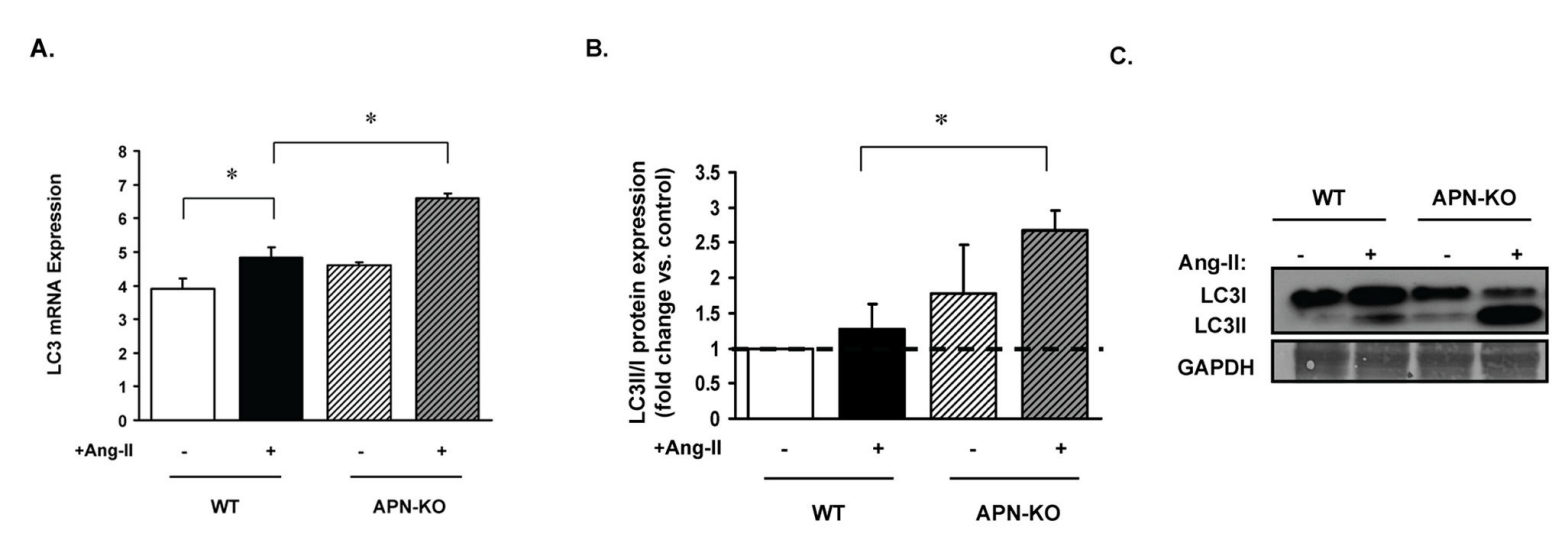
Loss of APN enhances autophagy in response to Ang-II infusion in vivo. WT and APN-KO mice were infused with Ang-II (3.2mg/kg/d) for 14 days, and LC3 gene and LC3II/I protein expression ratio was assessed from the LV of the hearts. (**A**) LC3 gene expression was increased in 37±1.1% in Ang-II infused APN-KO mice vs. WT Ang-II infused mice (*p<0.05) (**B**) LC3II/I protein expression was increased in Ang-II infused APN-KO mice by a factor of 2.7±0.6 vs. WT Ang-II infused mice (*p<0.05). (**B**) Representative Western blot.

In the LV of Ang-II infused APN-KO and WT mice hearts, LC3II/I gene and protein expression ratio were determined. LC3II/I gene and protein ratio expression were increased in Ang-II infused APN-KO mice by 37±1.1% and a factor of 2.7±0.6, respectively vs. WT Ang-II infused mice (Figure 7A–C). There was no difference in myocardial beclin-1 expression between Ang-II infused APN-KO and WT mice (data not shown). Thus, loss of APN increased the LC3II/I ratio in response to Ang-II infusion *in vivo.*

## Discussion

In isolated adult cardiomyocytes (1) APN modulated H_2_O_2_-induced autophagic cell death. (2) H_2_O_2_ induced autophagy by phosphorylation and thus inhibiting mTOR. This signaling pathway was ameliorated by APN(3). H_2_O_2_-induced autophagy directly activated AMPK and ERK which is also negatively regulated by APN(4). H_2_O_2_ inhibition of Akt is independent of mTOR phosphorylation. Finally, in mice subjected to chronic Ang-II infusion and oxidative stress, loss of APN enhances myocardial LC3II/I protein expression ratio*.*

### ROS and autophagy

In order to maintain appropriate cardiac function, the heart must adapt to elevated cardiovascular stress and accumulation of ROS [21,27,29,48]. Increase ROS eventually overwhelms antioxidant defenses and lead to a state of oxidative stress [30], which may further impair cardiac function and result in clinical deterioration [26,50,51]. In this present study, we showed that H_2_O_2_ (1mM) decreased cardiomyocyte viability, whilst APN pretreatment partially protected against the loss of cell viability. The concentration-dependent effect of H_2_O_2_ did not show significant myocyte necrosis/autophagy at <1mM, thus 1mM was the chosen concentration. Therefore, H_2_O_2_ induces both hypertrophy [25] and autophagy (in the present study) in a concentration-dependent manner in ARVM. Taken together with our prior studies, we conclude that the effects of ROS on myocyte phenotype are determined, at least in part, by the concentration of ROS. Autophagy is highlighted by the absence of cell death under basal conditions, where cell function is maintained; whereas excessive autophagy causes cellular destruction and is referred to as type-II cell death [31]. Consistent with prior reports that autophagy is upregulated in response to ROS [33], our results show that pathophysiological concentrations of H_2_O_2_ induces autophagosome formation in isolated adult cardiomyocytes. We previously demonstrated that *physiological* concentrations of H_2_O_2_ induces a pro-hypertrophic phenotype that is abrogated by APN via an AMPK/ERK/NF-κB signaling pathway [25]. In the present study, we found that MAPK/ERK1/2 or AMPK pathways are activated at high concentrations of H_2_O_2_ and may initially play a protective role against H_2_O_2_-induced autophagy. These findings suggest that the regulation of autophagy by ROS involves multiple kinase signaling pathways [37]. However, since APN partially rescues this phenotype by ameliorating the AMPK-mTOR-(MAPK)/ERK signaling pathway, it is likely that MAPK/ERK1/2 or AMPK pathways are deleterious. ROS-induced autophagy in ARVM is independent of beclin-1. Although apoptosis and autophagy share common stimuli and signaling pathways, and exhibit some degree of mutual inhibition; it is possible that beclin-1 might be involved in H_2_O_2_-induced apoptosis [22] and not autophagy. Our findings highlight the protective actions of APN in response to pathological levels of oxidative stress in cardiomyocytes.

### Role of ERK in autophagy

Although some studies suggest that ERK plays a role in modulating autophagy [44,52,53]; ERK activation appears to have divergent roles in autophagy in different cell types. ERK induces autophagy in neuronal cell death [54] and cancer cells [55,56] and ERK upregulates starvation-induced autophagy by down-regulating Akt/mTOR/S6K [45]. Similarly Wang et al., proposed that a non-canonical MEK/ERK module regulates autophagy through an AMPK-MEK/ERK-TSC-mTOR signaling pathway [57]. Here mTOR regulates autophagy induced by starvation and non-starvation stimuli that activate MEK/ERK, suggesting a possible universal mechanism in autophagy regulation through mTOR. Thus, in our study pathophysiological levels of H_2_O_2_ induces an autophagic phenotype that is also mediated by ERK activation in addition to an AMPK-mTOR signaling pathway.

### Akt and autophagy

Our data show that although H_2_O_2_ decreases Akt expression it does not lead to subsequent mTOR inhibition, thus H_2_O_2_-induced autophagy is independent of Akt signaling in cardiomyocytes. Although Akt inhibition leads to mTORC1 inhibition and its inhibition promotes autophagy, it has been shown in cancer cells that subsequent downstream signaling of Akt can contribute to autophagic regulation independent of mTOR [58]. Akt inhibition induces increases in mitochondrial superoxide and cellular ROS signals that activate autophagy in cancer cells [58]. In our study, cardiomyocytes treated with APN alone or concomitantly with H_2_O_2_ showed no difference in the increased Akt phosphorylation. Thus Akt activation does not play a direct role in the amelioration or propagation of H_2_O_2_-induced autophagy in ARVM.

### Autophagy and cardiac remodeling

As we have previously shown, mice with APN deletion, subjected to chronic Ang-II infusion, demonstrated increased ROS, hypertension and LVH [25,48]. We thus utilized this model as an experimental model of ROS and diastolic HF. With cardiac hypertrophy and hypertension, LV systolic function was preserved and associated with increased myocardial autophagy. However the presence of autophagy does not establish a cause or effect in the mechanism of cardiac remodeling. Autophagy can be both deleterious [34,35] or protective in degenerative diseases [17,31,32]; thus its presence in cardiac remodeling may be an epiphenomenon and reflect the presence of adverse cardiac remodeling and LVH. For example, cardiac-specific loss of ATG5 resulted in LVH, left ventricular dilatation and contractile dysfunction in mice [34,35]. Conversely, the mere presence of autophagy may also reflect an “autophagic” attempt to modulate Ang-II induced ROS when the protective actions of APN are absent. Our study, however, does not establish if autophagy is a mechanism or an effect of cardiac remodeling. In fructose-fed mice increased myocardial autophagy was associated with systemic insulin resistance, elevated cardiac superoxide production and suppressed cell survival signaling [59]. The presence of autophagy in IFNγKO subjected to chronic aldosterone mice suggests that it may play a role in LV cardiac remodeling and diastolic dysfunction in hypertension-induced diastolic HF [60].

### Protective actions of APN

APN mediates anti-hypertrophic effects in cardiomyocytes [25] and protects against ischemia-reperfusion injury [47,61]. Conversely, lack of APN results in adverse cardiac remodeling and increased mortality compared to WT controls in experimental models of cardiovascular stress [8,25,47,61,62]. In colorectal cancer cells, APN promotes cell survival during glucose deprivation by AMPKα and PPARα activation and IGF-1/PI3k/Akt/mTOR pathway inhibition [32]. Consistent with our present findings, oxidative stress activates the ERK/MAPK pathway [22,25] and the AMPK pathway [63] in cardiomyocytes. Both signaling pathways may inhibit mTOR, ultimately leading to the downstream formation of autophagosomes [43,64,65]. Low dose, physiological H_2_O_2_ (10 µM) has no effect on AMPK phosphorylation and pretreatment with APN increased AMPK phosphorylation in cardiomyocytes [25]. Conversely, higher concentrations of H_2_O_2_ (1mM) increased AMPK phosphorylation and inhibited mTOR phosphorylation, thus increasing autophagy. Pretreatment of cardiomyocytes with APN decreased H_2_O_2_-induced AMPK phosphorylation. Although others have reported that APN causes AMPK activation in cultured rat cardiomyocytes [47,66], none of these studies involved H_2_O_2_. Activation of Akt can also lead to decreased AMPK activity [67,68]; thus it is possible that APN mediates Akt phosphorylation and decreases AMPK with resultant mTOR activation and inhibition of autophagy. It is therefore conceivable that APN activates AMPK under some conditions, whilst inhibiting it under other conditions such as elevated oxidative stress. In our study, H_2_O_2_-induced autophagy occurred through a predominant AMPK/mTOR/ERK pathway, which was inhibited by APN. We did not observe changes in beclin-1 levels similar to other cell systems [69–71] which showed beclin-1 independent induced autophagy. Thus beclin-1 is not directly involved in the signaling mechanism we propose here.

Other potential protective actions of APN against autophagy have been proposed, such as angiogenesis where APN administration increases VEGF expression [72] and induces vascularization. However the antioxidant potential of APN in directly suppressing ROS may be most important [25]. APN inhibits platelet aggregation by attenuating oxidative and nitrosative stress by inhibiting inducible nitric oxide synthase and superoxide production *in vivo* [66]. Furthermore, in an ischemia/reperfusion-injury porcine model, APN modulated ROS metabolite levels and increased antioxidant levels [73].

**In conclusion**, APN protects against oxidative-stress mediated autophagic-induced cardiac myocyte death by suppressing the autophagic machinery predominantly via an ERK-mTOR-AMPK signaling mediated pathway. Recently in an experimental model of chronic Ang–II stimulation, LVH, fibrosis, and left ventricular diastolic dysfunction were modulated by a mitochondrial targeted antioxidant peptide, SS-31 [74]. Thus targeting mitochondrial ROS may be a therapeutic option in patients with HF-preserved EF [74]. Our study suggests that these options may extend to APN or an APN-mimetic. Additional in-depth studies will be needed to further dissect cross-talk between other pathways. *In toto*, our present findings provide mechanistic insight into the anti-oxidant potential of APN and underscore its protection in cardiovascular diseases such as HF-preserved EF where a paucity of therapeutic interventions exists.

## Supporting Information

Figure S1 APN-attenuates H_2_O_2_-mediated p62 expression in ARVM. (**A**) 1mM H_2_O_2_ (6hr) increased p62 protein expression ratio in ARVM by a factor of 4.0±0.5 (**p<0.01 vs. control). This was abrogated by pretreatment with APN (54±4% reduction; **p<0.01 vs. H_2_O_2_-treated cells). (**B**) Representative Western blot.
(TIFF)

Figure S2 H_2_O_2_ caused a decrease in phospho-Akt mRNA independepent of mTOR. (**A**) 1mM H_2_O_2_ (15min) decreased phospho-Akt gene expression in ARVMs by 21±2% (*p<0.05 vs. control). APN alone and pretreatment with APN significantly increased phospho-Akt gene expression (‡p<0.001 vs. control, for both). (**B**) Transduction with β-gal had no effect on mTOR mRNA in control cells. H_2_O_2_ decreased mTOR mRNA (*p<0.05 vs. control). Transduction with dn-Akt had no effect on control cells or on the H_2_O_2_-induced mTOR decrease in gene expression (1.0±0.01 in β-gal), 0.64±0.10 in β-gal + H_2_O_2_, 0.99±0.04 in dn-Akt, 0.65±0.06 in dn-Akt + H_2_O_2_.
(TIF)
