# Supplementary figures and images for: Adiponectin Modulates Oxidative Stress-Induced Autophagy in Cardiomyocytes
Source: PLoS One. 2013 Jul 19;8(7):e68697. doi: 10.1371/journal.pone.0068697 (PMC3716763; doi:10.1371/journal.pone.0068697)

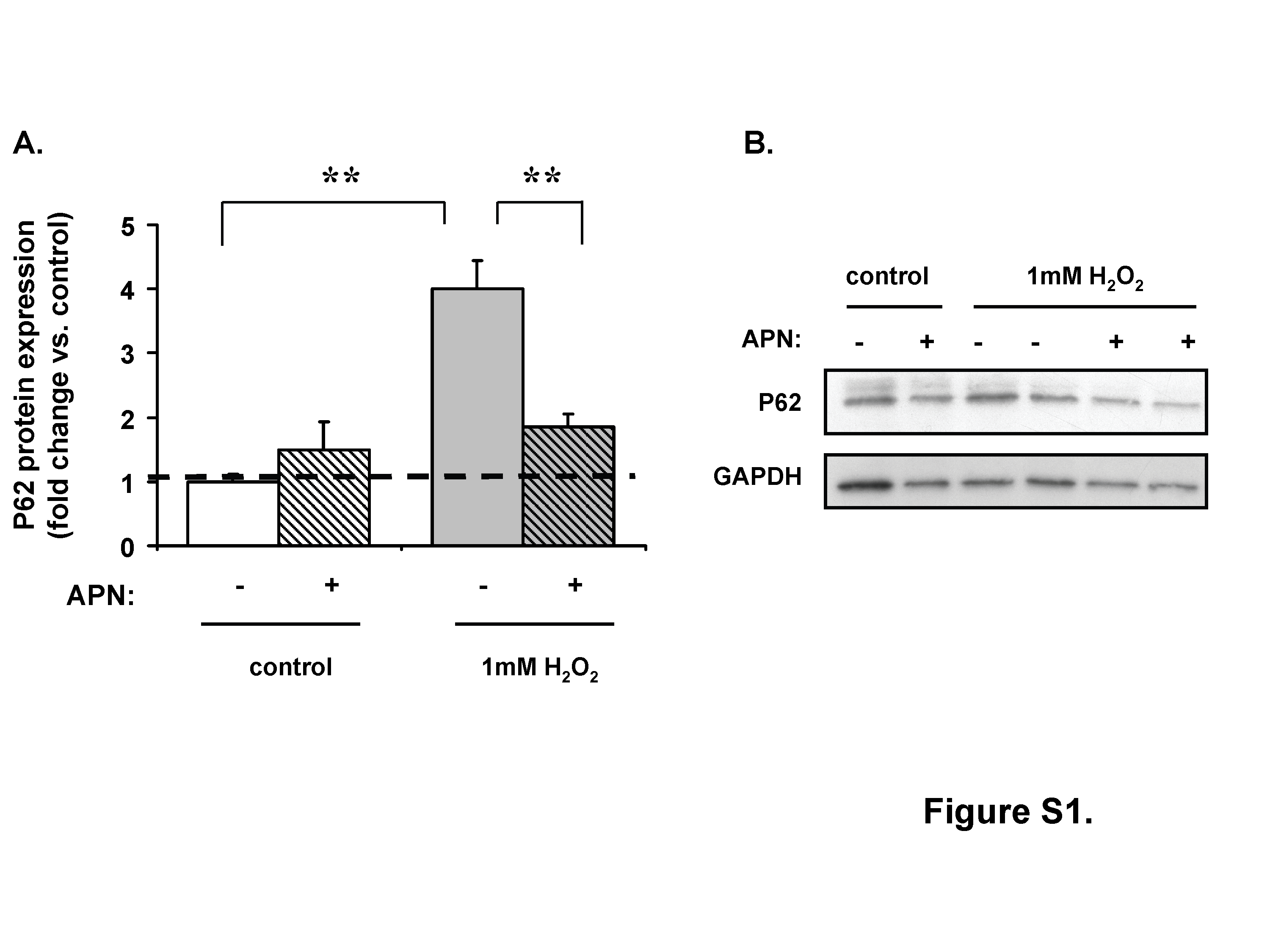

Supplement: Figure S1 — (A) 1mM H2O2 (6hr) increased p62 protein expression ratio in ARVM by a factor of 4.0±0.5 (**p<0.01 vs. control). This was abrogated by pretreatment with APN (54±4% reduction; **p<0.01 vs. H2O2-treated cells). (B) Representative Western blot. (TIFF) [file pone.0068697.s001.tiff]

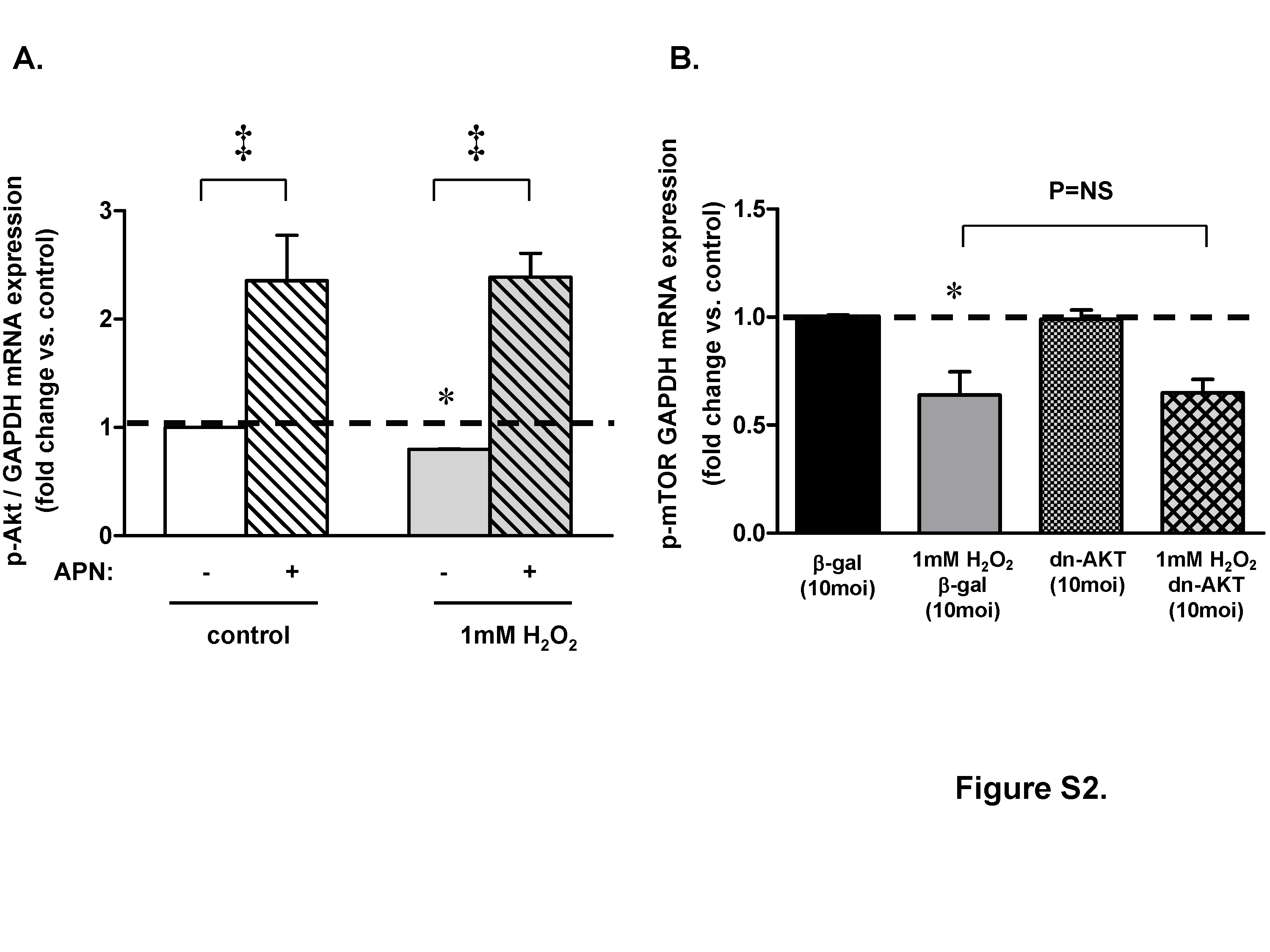

Supplement: Figure S2 — (A) 1mM H2O2 (15min) decreased phospho-Akt gene expression in ARVMs by 21±2% (*p<0.05 vs. control). APN alone and pretreatment with APN significantly increased phospho-Akt gene expression (‡p<0.001 vs. control, for both). (B) Transduction with β-gal had no effect on mTOR mRNA in control cells. H2O2 decreased mTOR mRNA (*p<0.05 vs. control). Transduction with dn-Akt had no effect on control cells or on the H2O2-induced mTOR decrease in gene expression (1.0±0.01 in β-gal), 0.64±0.10 in β-gal + H2O2, 0.99±0.04 in dn-Akt, 0.65±0.06 in dn-Akt + H2O2. (TIF) [file pone.0068697.s002.tif]
